# Genome-Wide Identification, Characterization and Expression Analysis of TCP Transcription Factors in *Petunia*

**DOI:** 10.3390/ijms21186594

**Published:** 2020-09-09

**Authors:** Shuting Zhang, Qin Zhou, Feng Chen, Lan Wu, Baojun Liu, Fei Li, Jiaqi Zhang, Manzhu Bao, Guofeng Liu

**Affiliations:** 1 Key Laboratory of Horticultural Plant Biology, Ministry of Education, College of Horticulture and Forestry Sciences, Huazhong Agricultural University, Wuhan 430070, China; shutingzh@webmail.hzau.edu.cn (S.Z.); zhouqin@webmail.hzau.edu.cn (Q.Z.); chenf@webmail.hzau.edu.cn (F.C.); ivylanblue@126.com (L.W.); bjliumail@126.com (B.L.); Lifei8711@webmail.hzau.edu.cn (F.L.); jiaqizhang@mail.hzau.edu.cn (J.Z.); 2 Guangzhou Institute of Forestry and Landscape Architecture, Guangzhou 510405, China

**Keywords:** petunia, TCP transcription factors, expression patterns, petal development, floral organ size

## Abstract

The plant-specific TCP transcription factors are well-characterized in both monocots and dicots, which have been implicated in multiple aspects of plant biological processes such as leaf morphogenesis and senescence, lateral branching, flower development and hormone crosstalk. However, no systematic analysis of the petunia *TCP* gene family has been described. In this work, a total of 66 petunia *TCP* genes (32 *PaTCP* genes in *P. axillaris* and 34 *PiTCP* genes in *P. inflata*) were identified. Subsequently, a systematic analysis of 32 *PaTCP* genes was performed. The phylogenetic analysis combined with structural analysis clearly distinguished the 32 *PaTCP* proteins into two classes—class Ι and class Ⅱ. Class Ⅱ was further divided into two subclades, namely, the CIN-TCP subclade and the CYC/TB1 subclade. Plenty of *cis*-acting elements responsible for plant growth and development, phytohormone and/or stress responses were identified in the promoter of *PaTCPs*. Distinct spatial expression patterns were determined among *PaTCP* genes, suggesting that these genes may have diverse regulatory roles in plant growth development. Furthermore, differential temporal expression patterns were observed between the large- and small-flowered petunia lines for most *PaTCP* genes, suggesting that these genes are likely to be related to petal development and/or petal size in petunia. The spatiotemporal expression profiles and promoter analysis of *PaTCPs* indicated that these genes play important roles in petunia diverse developmental processes that may work via multiple hormone pathways. Moreover, three PaTCP-YFP fusion proteins were detected in nuclei through subcellular localization analysis. This is the first comprehensive analysis of the petunia *TCP* gene family on a genome-wide scale, which provides the basis for further functional characterization of this gene family in petunia.

## 1. Introduction

The final shape and size of lateral organs such as leaves and flowers are established mainly by the elaborate coordination of cell proliferation and cell expansion in response to internal developmental and external environmental cues. This coordination relies on the complex regulatory circuits in which many microRNAs (miRNAs) and transcription factors are recruited [1]. The *TCP* gene family, first described in 1999 and named after the initials of four characterized members, namely Teosinte Branched 1 (TB1) from maize (*Zea mays*), CYCLOIDEA (CYC) from snapdragon (*Antirrhinum majus*) and PROLIFERATING CELL FACTORS (PCF1 and PCF2) from rice (*Oryza sativa*), encompasses a group of phylogenetically related and plant-specific transcription factors (TFs). TCP proteins are featured by a highly conserved non-canonical basic helix–loop–helix (bHLH) DNA-binding domain referred to as the TCP domain, which is responsible for the mediation of DNA binding, nuclear localization as well as interactions with other proteins [2]. Based on the differential features within the conserved TCP domain, TCP proteins can be categorized into two classes: class I (or TCP-P clade) and class II (or TCP-C clade), and the latter can be further divided into the ubiquitous CINCINNATA (CIN-TCP subclade) and the angiosperm-specific CYC/TB1 subclade [3,4]. Different from class II TCPs, the class I TCP proteins possess a four-amino-acid deletion in the basic region within the TCP domain. Besides the conserved TCP domain, some class II members contain an arginine-rich R domain with unknown biological function, and the majority of the CYC/TB1 subclade members have a conserved ECE motif (a glutamic acid-cysteine-glutamic acid stretch) that remains functionally uncharacterized [3,5].

Accumulating evidence indicates that TCP transcription factors have versatile functions in a plethora of plant developmental processes, such as lateral branching, leaf development and senescence, flowering and flower development, gametophyte development, seed germination and defense responses [6,7,8,9,10,11,12,13,14,15,16]. The crosstalk between TCP transcription factors and hormone signaling as well as their roles in mediating the hormone activity in cell proliferation has been reviewed in detail [17]. As for class II TCP, five *Arabidopsis CIN*-like genes (*TCP2-4*, *10* and *24*), which are targeted by *miR319*, have been shown to participate in the transcriptional control of cell cycle genes during leaf development and morphogenesis. Overexpression of *miR319* or downregulation of TCP genes induce excess cell proliferation, thus leading to crinkled leaves [8,10,18]. Three nontargeted *TCP5*-like genes are demonstrated to promote the transition from cell division to post-mitotic cell expansion during petal development [19]. Moreover, *Arabidopsis* CIN-TCPs have been implicated in the regulation of floral organ development and flowering time [20,21]. Recent advances in the understanding of the function and regulation of CIN-like *TCP* genes have been summarized by Qin et al. [22]. To our knowledge, the CYC/TB1 clade mainly plays a prominent role in axillary meristem development [3]. *AtTCP18* and *AtTCP12,* two homologs of maize *TB1*, are identified as integrators of branching signals and negatively regulate the axillary bud outgrowth [6,23]. AtTCP18 also engages in suppressing the precocious floral transition of the axillary meristems by interacting with florigen proteins [24]. *AtTCP1*, the homolog of *CYC*, is associated with brassinosteroid (BR) biosynthesis and the longitudinal elongation of leaves [25,26].

By contrast, class I TCPs are suggested to promote cell proliferation and plant growth [3]. Most loss-of-function mutants of class I TCP genes exhibit mild or no defective phenotypes due to genetic redundancy [9]. Even so, several class I TCP transcription factors have been integrated into multiple hormone-signaling pathways and participated in the regulation of inflorescences stem elongation [27], gynoecium development [28], seed germination [15], filament elongation of stamen [29] and flowering [13] in *Arabidopsis*. AtTCP20 binds to the GCCCR motif of the *CYCB1* promoter and participates in the regulation of cell division, cell expansion and growth coordination [30]. Given that both classes of TCP proteins have distinct but overlapping DNA binding sites: GGNCCC [31], it has been reported that class I and II TCP proteins participate in antagonistic and synergistic biological interactions [32]. For example, *AtTCP15* indirectly regulates the boundary-specific genes through the auxin pathway, which partly overlaps with the one affected by CIN-TCP genes [33,34]. *AtTCP20* and its direct target *AtTCP9* repress the jasmonate acid (JA) biosynthesis, which is antagonized by the CIN-TCP protein TCP4 [10,35].

To date, the *TCP* gene family has been identified and characterized in various plant species, such as *Arabidopsis* and rice [36], tomato [37], *Prunus mume* [38], watermelon [39], strawberry [40], poplar [41], turnips [42], Chinese cabbage [43], *Medicago* [44], chickpea [45], switchgrass [46] and grapevine [47]. Petunia is well-known as a popular and important ornamental plant belonging to the Solanaceae family. Considering the advantages of the highly efficient genetic transformation system, ease of cultivation and propagation as well as genetic diversity, petunia has been used as a genetic model system for a long history [48,49]. For ornamental plants, flower size is an important trait and is always the breeding objective of horticultural breeders. Recently, the whole-genome sequencing and assembly of two wild parents of petunia, *P. axillaris* and *P. inflata*, was achieved [50], which contribute significantly to identify and characterize *TCP* genes in petunia on a genome-wide scale. However, only three petunia *CYC/TB1* subclade genes (*PhTCP1-PhTCP3*, isolated from *Petunia hybrida* inbred line V26) are functionally characterized as the regulators of the strigolactone (SL) signaling pathway [51]; no comprehensive analysis of the petunia *TCP* gene family was reported. In this study, a genome- and transcriptome-wide analysis of the *TCP* gene family in petunia was carried out for the first time. The present work identified 32 *PaTCP* genes from *P. axillaris*, together with analyzing their gene classification, phylogenetic relationships, conserved motifs, exon-intron organization, putative promoter *cis*-acting elements, as well as subcellular localization. In addition, the spatiotemporal expression profiles of *PaTCP* genes were investigated by real-time quantitative RT-PCR (qPCR). This study lays a good basis for further exploring the potential roles of *PaTCP* genes in plant growth and development.

## 2. Results

### 2.1. Identification of TCP Genes in Petunia Species

To identify the petunia *TCP* genes, *Arabidopsis* and tomato TCP protein sequences as well as the HMM profile of the TCP domain (PF03634) were used to search against the genome and transcriptome datasets of *P. axillaris* and *P. inflata* [50,52]. As a result, 32 *PaTCP* genes were identified in *P. axillaris* (Table 1, Table S1) and 34 *PiTCP* genes were identified in *P. inflata* (Table S2). We named these petunia *TCP* genes according to the closest homologs of them in *Arabidopsis*. The number of TCP genes in *P. axillaris* (32 members) and *P. inflata* (34 members) is almost 1.3 and 1.4 times more, respectively, than that in *Arabidopsis* (24 members), which is consistent with protein-coding genes in the *P. axillaris* genome (32,928) and *P. inflata* genome (36,697) compared to the *Arabidopsis* genome (25,498) [50,53].

Sequence analysis demonstrated that the length of *PaTCP* genes varied from 624 bp (*PaTCP11*) to 1698 bp (*PaTCP18b*) and the ORF length of *PaTCPs* varied from 624 bp (*PaTCP11*) to 1572 bp (*PaTCP8*); the mean length of the 32 deduced PaTCP proteins was 346 amino acids (range 207-523 amino acids) (Table 1, Table S1). Similarly, the length of *PiTCP* genes varied from 624 bp (*PiTCP11*) to 1584 bp (*PiTCP18c*), the ORF length of *PiTCPs* varied from 624 bp (*PiTCP11*) to 1572 bp (*PiTCP8*), and the size of deduced PiTCP proteins ranged from 207 to 523 amino acids with an average of 350 amino acids (Table S2). More sequence characteristics of PaTCPs and PiTCPs, such as the isoelectric point (pI), molecular weight (MW) and the location at *Petunia* scaffolds, are listed in Table 1 and Table S2, respectively.

### 2.2. Phylogenetic Analysis of TCP Proteins from Petunia, Tomato and Arabidopsis

**Figure 1:**
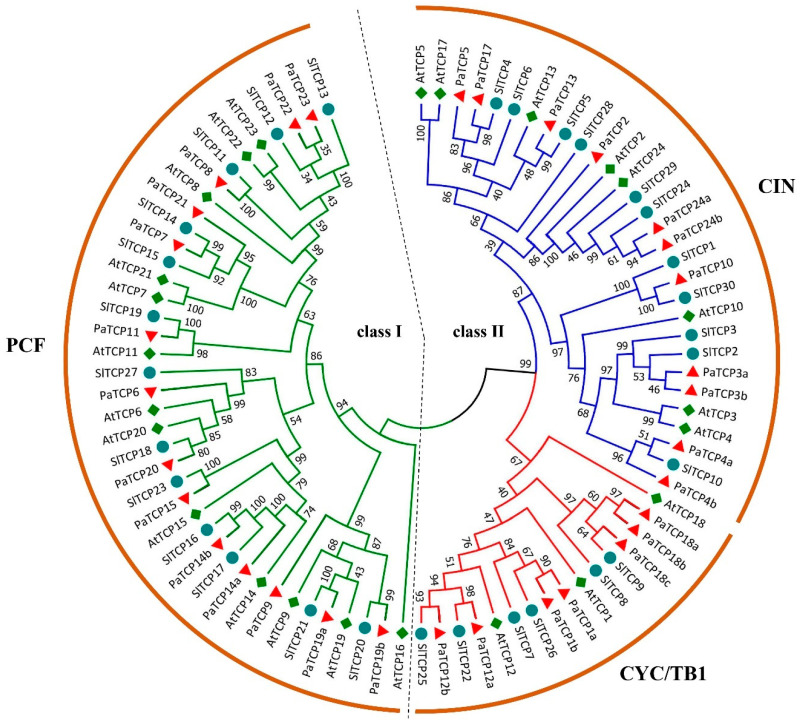
Phylogenetic relationship of TCP transcription factors from petunia, *Arabidopsis* and tomato, based on the multiple alignments of the full-length amino acid sequences of TCP proteins from *P. axillaris* (PaTCP), tomato (SlTCP) and *Arabidopsis* (AtTCP). The neighbor-joining (NJ) algorithm-based phylogenetic tree was built using MEGA7.0 software with 500 bootstrap replicates. Green, red and blue lines represent the PCF, CYC/TB1 and CIN subclades, respectively.

**Figure 2:**
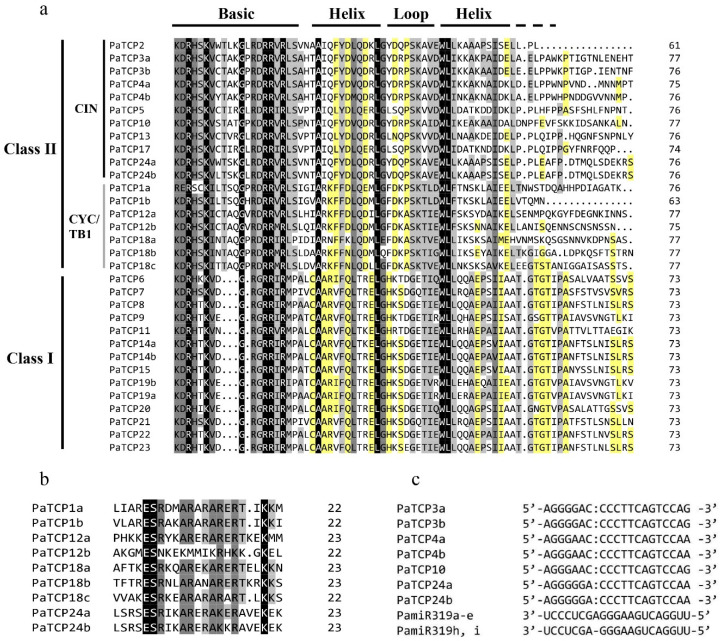
Multiple sequence alignments of PaTCP proteins and analysis of the putative *miR319* binding sites. (**a**). Alignment of the TCP domain and adjoining sequence for the predicted PaTCP proteins. Overall conserved amino acids were shaded in black. The Basic, Helix I, Loop and Helix II regions are indicated. (**b**). Alignment of the R-domain of class II PaTCP members. (**c**). Alignment of putative target areas for PamiR319 (aligned in reverse).

To clarify the evolutionary and phylogenetic relationships between the PaTCP proteins and other known TCPs, an unrooted phylogenetic tree was generated with the neighbor-joining (NJ) method using the full-length sequences of 32 PaTCP proteins together with 24 *Arabidopsis* TCP proteins (AtTCPs) and 30 tomato TCP proteins (SlTCPs) (Figure 1). Multiple alignments of the conserved TCP domains of 32 PaTCPs were also performed (Figure 2a). Both the phylogenetic analysis and multiple alignments of the TCP domains suggested that these PaTCP proteins were classified into two classes: 14 of 32 PaTCPs belong to class I (PCF/TCP-P clade) because of a deletion of four amino acids in the basic region, and 18 belong to class II (TCP-C clade) which could be further divided into the CYC/TB1 subclade and CIN subclade (Figure 1 and Figure 2a). The class I PaTCPs encompassed a group of relatively closely related proteins with extended homology from the TCP domain to C-terminal. In addition, the key residues in the basic region exhibited high conservation, while the residues in the loop and the hydrophilic residues of the helices were relatively less conserved in both classes I and II PaTCPs (Figure 2a). Notably, PaTCP1a protein lacked the conserved residues of the basic region at the N-terminus, suggesting that this protein may have a deficiency of DNA binding ability.

Except for the TCP domain, eight class II proteins, PaTCP1a, PaTCP1b, PaTCP12a, PaTCP18a, PaTCP18b, PaTCP18c, PaTCP24a and PaTCP24b, shared the R domain (18-residues arginine-rich motif) at the C-terminus downstream of the TCP domain, and an R domain-like sequence was present in PaTCP12b, but less conserved (Figure 2b). Additionally, seven *PaTCPs* (*PaTCP3a*, *3b*, *4a*, *4b*, *10*, *24a* and *24b*) contained the putative *miR319* target sites (Figure 2c). However, *PaTCP2*, the closest petunia homolog of *AtTCP2*, had no *miR319* target site due to a nonsense mutation just after the TCP domain.

### 2.3. Gene Structure and Conserved Motif Analysis

**Figure 3:**
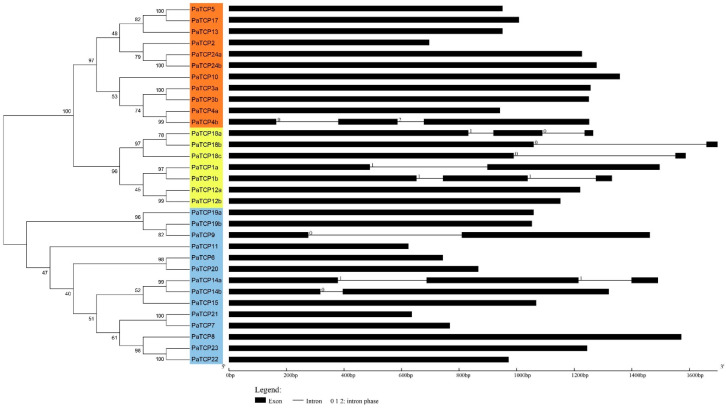
Exon-intron structures of *PaTCP* genes. The cladogram was produced by MEGA 7.0 software with the NJ method using the full-length sequences of PaTCP proteins. The orange, yellow and blue rectangles were used to cluster the genes into the CIN, CYC/TB1 and PCF subclades, respectively. 5′UTR and 3′UTR were not shown. The exons and introns are represented by a black block and a thin gray line, respectively. The 0, 1 and 2 denote intron phases.

To further explore the structural features of petunia *TCP* genes, the exon-intron organization was analyzed. The class II *PaTCP* genes contained more introns than those of class Ι genes (Figure 3). Among the class I *PaTCP* genes, 11 of 14 had no intron and the other three genes had one or two introns. For CIN subclade genes, only *PaTCP4b* contained two introns and all the others had no intron. In contrast, most CYC/TB1 subclade genes possessed one or more intron, with the exception of *PaTCP12a* and *PaTCP12b*, which had no intron. Moreover, similar exon/intron structures were also observed in *PiTCPs* (Figure S1).

**Figure 4:**
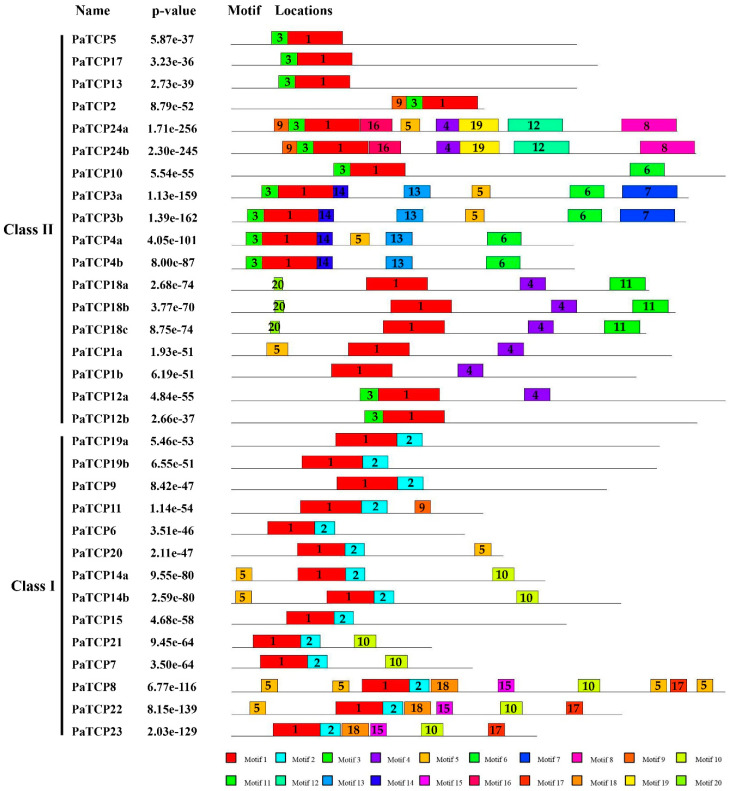
Distribution of the conserved and potential motifs of PaTCP proteins. Each motif is represented by rectangular boxes with different colors and numbered from 1 to 20.

To get more understanding of the structural and functional divergences of the PaTCP proteins, the conserved motif compositions of PaTCPs were investigated using the online MEME tool. As shown in Figure 4, a total of 20 motifs were identified and designated as motif 1 to motif 20. The number of motifs varied from 2 to 10. Three motifs (motif 1, 2 and 3) were identified as the TCP domain, and motif 1 was uniformly observed in all PaTCP proteins. Some motifs only presented in a specific subclade. For instance, all class I proteins shared motif 2 in the C-terminal TCP domain, while motif 3 of the N-terminal TCP domain was conserved in all CIN subclade members. In addition to the TCP domain, motif 4, identified as the conserved R domain, was hit in six CYC/TB1 proteins and two CIN subclade proteins. Moreover, some motifs were exclusively present in specific subfamily members, such as motif 6, 7, 8, 12, 13 and 14 in the CIN-TCP subclade, motif 11 and 20 in CYC/TB1 subclade and motif 10, 15, 17 and 18 in class I members. Similar motif compositions were observed in PaTCP proteins, which were clustered into the same clade or subclade, while significant divergences were found in different subclades (Figure 4), suggesting that PaTCP proteins likely have functional divergence between subclades and functional redundancy within the subclade.

### 2.4. Expression Profiles of PaTCP Genes

**Figure 5:**
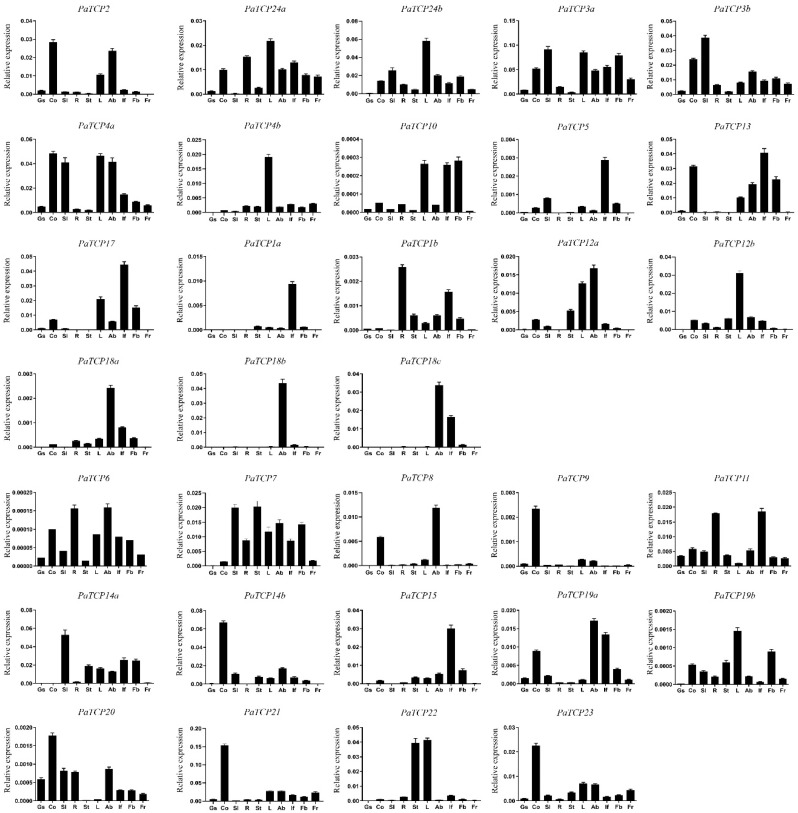
Expression analysis of *PaTCP* genes by qPCR. Gs, germinating seeds; Co, cotyledons; Sl, young seedlings; R, roots; St, stems; L, leaves; Ab, axillary buds; If, inflorescences; Fb, flower buds (0.5 cm); Fr, fruits. The relative expression level was normalized to the petunia *EF1α* gene. For each tissue, three biological replicates were used to calculate the mean values ± SD (standard deviation) with the 2^−ΔΔCT^ method.

In order to predict the potential function for *PaTCP* genes, we examined the spatial expression profiles of 32 *PaTCP* genes in various tissues, including germinating seeds, cotyledons, seedlings, roots, stems, leaves, axillary buds, inflorescences, flower buds and fruits by qPCR. As indicated in Figure 5, the expression patterns of *PaTCP* genes could be classified into four groups. The first group was ubiquitously expressed in almost all tested tissues, including *PaTCP3a*, *PaTCP3b*, *PaTCP4a*, *PaTCP7*, *PaTCP11*, *PaTCP19a*, *PaTCP21*, *PaTCP23, PaTCP24a* and *PaTCP24b*. Of these, *PaTCP3a*, *PaTCP3b*, *PaTCP4a, PaTCP24a* and *PaTCP24b* showed relatively high levels of expression in most tissues with lower expression in germinating seeds, stems and roots or seedlings. *PaTCP11* showed the highest expression in inflorescences and roots. *PaTCP21* and *PaTCP23* showed the highest expression in cotyledons. The second group, including *PaTCP1b, PaTCP5*, *PaTCP9, PaTCP18a, PaTCP19b* and *PaTCP20,* especially *PaTCP6* and *PaTCP10,* showed a very low expression level (less than 0.005 relative to that of the reference gene *PhEF1α*) in all tissues (Figure 5). Among these genes, *PaTCP5*, *PaTCP9* and *PaTCP18a* showed more predominant expression in inflorescences, cotyledons and axillary buds, respectively. The third group exhibited a tissue-preferential expression pattern, including *PaTCP1a*, *PaTCP4b*, *PaTCP8*, *PaTCP12b*, *PaTCP14b, PaTCP15*, *PaTCP18b*, *PaTCP18c, PaTCP21* and *PaTCP22*. For example, *PaTCP1a* and *PaTCP15* showed a significantly higher level of expression in inflorescences. *PaTCP4b* and *PaTCP12b* were predominantly expressed in leaves, while *PaTCP8*, *PaTCP18b* and *PaTCP18c* were mainly detected in axillary buds. The transcript of *PaTCP22* was mostly detected in stems and leaves, while *PaTCP14b* was dominantly expressed in cotyledons. The fourth group, including *PaTCP2*, *PaTCP12a*, *PaTCP13*, *PaTCP14a* and *PaTCP17,* was expressed specifically in partial tissues. For instance, *PaTCP2*, *PaTCP13*, *PaTCP14a* and *PaTCP17* showed higher expression in cotyledons, inflorescences, flower buds and axillary buds, with very weak or no expression in germinating seeds, seedlings, roots, stems and fruits. *PaTCP12a* had the highest expression in axillary buds followed by leaves and stems.

### 2.5. Identification of Cis-Acting Elements in the Promoter of PaTCP Genes

**Figure 6:**
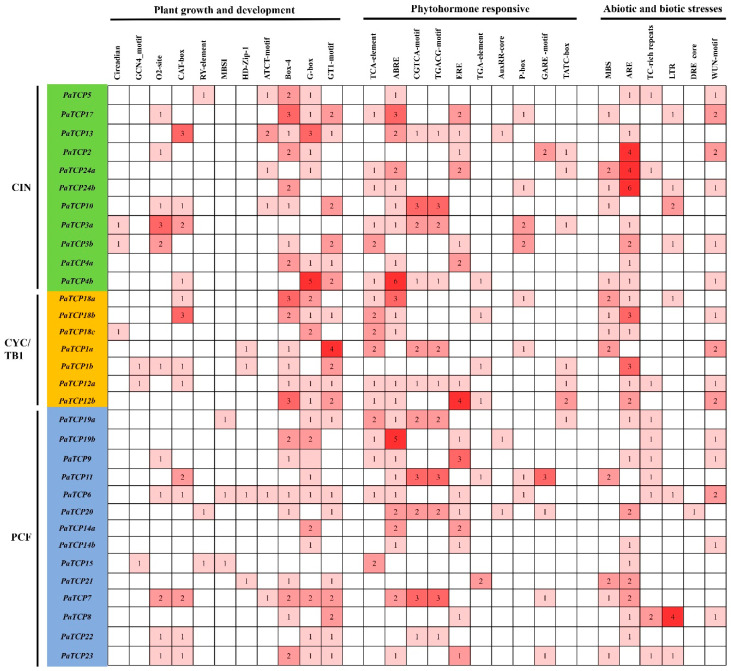
*Cis*-regulatory elements analysis of *PaTCP* promoters. Based on the functional annotation, the *cis*-acting elements were categorized into three major classes: plant growth and development, phytohormone responsive or abiotic and biotic stresses-related *cis*-acting elements (details are shown in Table S3).

To investigate the potential regulatory mechanism of *PaTCP* genes, the promoter regions (1.5 kb of genomic DNA sequence upstream of the translational start site) of the *PaTCPs* were submitted to the PlantCARE database [54]. Apart from the common CAAT-box and TATA-box, a large number of *cis*-elements responsible for plant growth and development, phytohormone responses, as well as abiotic and biotic stress responses were identified (Figure 6). The zein-metabolism regulation element (O2 site) and meristem expression and specific activation element (CAT-box) were found in 11 and 13 *PaTCP* genes, respectively. Additionally, some cis-regulatory elements related to plant growth and development, including leaf development-related element (HD-Zip1), the circadian control element (circadian), the endosperm-specific expression element (GCN4_motif), the flavonoid biosynthetic (MBSI), as well as the seed-specific regulation element (RY element), were also identified in four, three, three, three and three *PaTCP* genes, respectively. Meanwhile, many regulatory elements involved in light responsiveness were detected, such as ATCT-motif, Box-4, G-box and GT1-motif. In hormone-related cis-acting elements, the ABA-responsive element (ABRE), the SA-responsive element (TCA element) and the ethylene-responsive element (ERE) were frequently identified in the promoter regions of 24, 17 and 16 *PaTCP* genes, respectively. The gibberellin-responsive elements (GARE-motif, P-box and TATC-box) and MeJA-responsive elements (CGTCA-motif and TGACG-motif) were observed in the promoter regions of 17 and 11 *PaTCP* genes, respectively. Auxin-responsive elements (TGA element and AuxRR-core) were also found in the promoter regions of nine *PaTCP* genes. Additionally, some stress-related *cis*-acting elements such as drought-inducibility (MBS), anaerobic induction (ARE), low-temperature (LTR) and wounds (WUN-motif) were also detected in the promoter regions of *PaTCP* genes, which indicated that these *PaTCP* genes were likely to participate in defense signaling.

### 2.6. Transcriptional Profiles of PaTCPs during Petal Development in Large- and Small-Flowered Lines 

To gain an in-depth knowledge of the potential roles of *PaTCP* genes during petal development, we carried out qPCR to further investigate the transcript accumulation patterns of *PaTCPs* at five different petal developmental stages (S1–S5). These stages cover the developmental range from young flower bud to fully blooming flower. Samples were taken from the corollas of large- and small-flowered lines ‘L’ and ‘S,’ respectively, which originated from the self-cross separation of the same inbred line. The mean size of flower diameter of ‘L’ (8.53 ± 0.18 cm) is much larger than that of ‘S’ (3.54 ± 0.13 cm) (Figure S2). All 32 *PaTCP* genes were detected during petal development in both large- and small-flowered lines.

**Figure 7:**
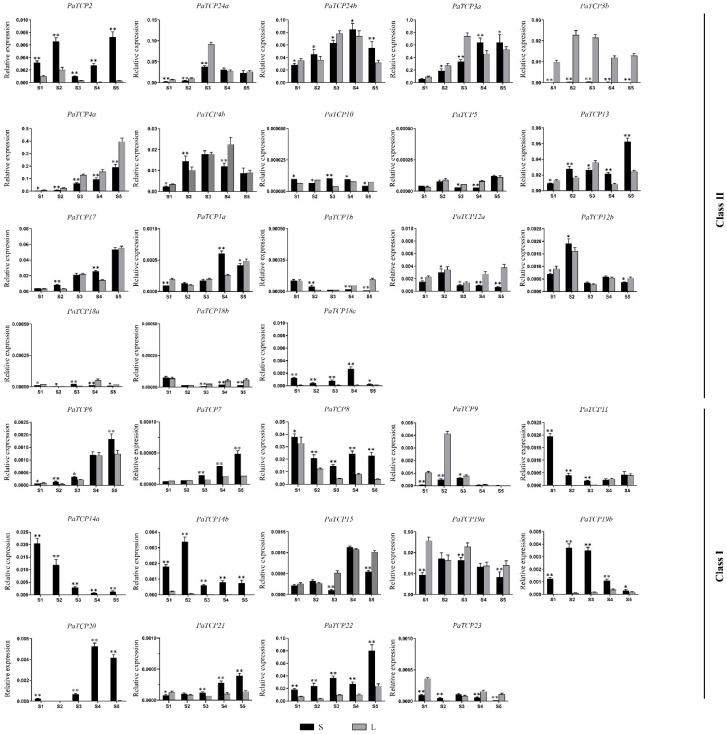
Transcriptional profiles of *PaTCP* genes in the large- and small-flowered lines, ‘L’ and ‘S,’ respectively, during different petal developmental stages: young flower buds (<0.5 cm, S1), extending flower buds (when flower buds just enclosed by sepals, S2), pre-anthesis (when flower buds extended to full length, S3), semi-open flowers (S4) and fully blooming flowers before the anthers dehisced (S5). The results were normalized to the expression of the petunia *EF1α* gene. The mean values ± SD (standard deviation) were calculated from three biological replicates with the 2^−ΔΔCT^ method. The black and dark grey square represented the ‘S’ and ‘L,’ respectively. Asterisks denote statistically significant differences between ‘S’ and ‘L’ at each developmental stage, as determined by Student’s *t*-test (* *p* < 0.05, ** *p* < 0.01).

As shown in Figure 7, seven *PaTCP* genes were gradually upregulated during the petal development in ‘L’ and/or ‘S,’ showing the highest expression in fully blooming corollas (S5). Of these, three class II *TCP* genes (*PaTCP3a*, *PaTCP4a* and *PaTCP17*) and four class I TCP genes (*PaTCP6*, *PaTCP7*, *PaTCP21* and *PaTCP22*) were included. On the contrary, several *PaTCPs,* such as *PaTCP8*, *PaTCP11*, *PaTCP14a, PaTCP19a* and *PaTCP23,* showed downregulated patterns, with the highest expression in young flower buds (S1 or S2). Moreover, the expression of *PaTCP4b*, *PaTCP15*, *PaTCP20*, *PaTCP24a* and *PaTCP24b* increased first and then dropped at later stages, with the highest expression in semi-open (S4) or fully blooming (S5) corollas. *PaTCP3b*, *PaTCP9*, *PaTCP12b* and *PaTCP14b* shared similar expression trends, but with the highest expression in young flower buds (S2). However, some *PaTCP* genes, including *PaTCP1a/b*, *PaTCP5*, *PaTCP10*, *PaTCP12a* and *PaTCP18a/b/c*, showed very low expression levels during all the petal developmental stages, and no regular trend was observed. These results suggested that most *PaTCP* genes should participate in the regulation of petal development in petunia.

Some *PaTCP* genes showed differential expression patterns between the large- and small-flowered lines during petal development process (Figure 7, Figure S3). The expression levels of eight genes (*PaTCP2*, *PaTCP8*, *PaTCP14a*, *PaTCP14b*, *PaTCP18c*, *PaTCP19b*, *PaTCP20* and *PaTCP22*) were significantly higher in ‘S’ than in ‘L’ during petal development. Among them, most genes belonged to the TCP-P clade. However, three genes (*PaTCP3b*, *PaTCP4a* and *PaTCP9*) exhibited a relatively higher expression in ‘L’ than in ‘S.’ For *PaTCP3b,* high levels of expression were observed in ‘L,’ while almost no transcript was detected in ‘S’ at various petal developmental stages.

### 2.7. Subcellular Localization of PaTCP Proteins

**Figure 8:**
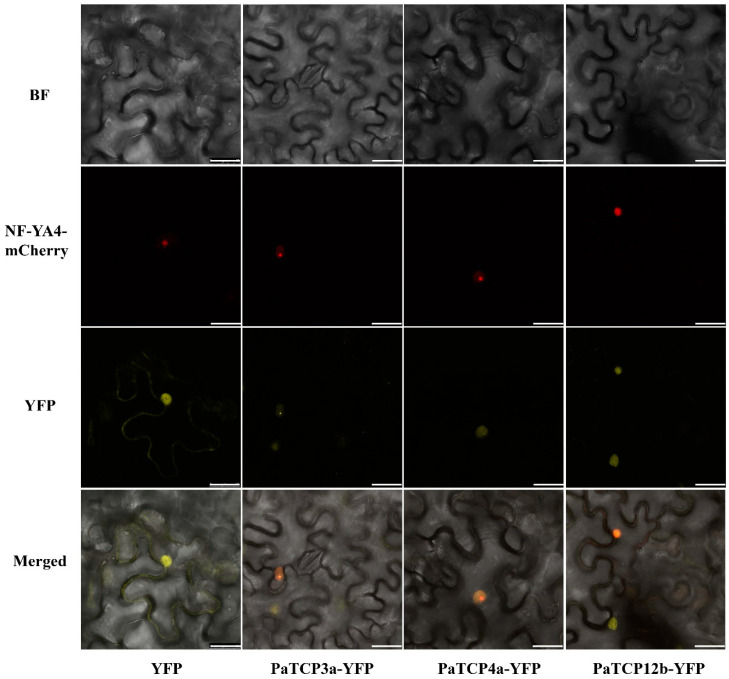
Subcellular localization of three PaTCP-YFP fusion proteins in tobacco. Empty vector of yellow fluorescence (YFP) was used as control. The co-transformation of the nuclear marker (NF-YA4-mCherry) was used to visualize the nuclei. Merged panel showed the nuclear localization in tobacco epidermis cells. Scale bar, 25 μm.

It has been reported that TFs are usually involved in regulating the transcription of target genes by binding to specific *cis*-elements in their promoters. We further examined the subcellular localization of three PaTCP proteins. The full-length coding sequences without the stop codon of three cloned *PaTCP* genes (*PaTCP3a*, *PaTCP4a* and *PaTCP12b*) were inserted into a vector with yellow fluorescence protein (YFP) under the control of the CaMV 35S promoter. Yellow fluorescence signals of these PaTCP-YFP fusion proteins were detected in the nuclei, which was consistent with the putative prediction of transcription factor activities of the PaTCP proteins (Figure 8).

## 3. Discussion

### 3.1. Evolution of TCP Genes in P. axillaris

The plant-specific TCP transcription factors have been implicated in exerting regulatory roles in multiple aspects of physiological and biological processes during plant growth and development [32]. In this study, we identified 32 *PaTCPs* from *P. axillaris* genome and 34 *PiTCPs* from *P. inflata* genome. The phylogenetic analysis and sequence alignment showed that all 32 PaTCPs were classified into three major groups: PCF-TCPs, CIN-TCPs and CYC-TCPs (Figure 1 and Figure 2a). The conserved TCP domain was shared by all PaTCP members, suggesting that PaTCP transcription factors possess the capacity of dimerization and DNA binding [2]. Moreover, all *Arabidopsis* and tomato TCPs were clustered into the same clade or subclade as reported previously [36,37]. Furthermore, most PaTCP members within the same clade and/or subclade display a similar exon-intron organization in terms of exon length and intron number (Figure 3), and relatively conserved motif compositions (Figure 4), which further support the close evolutionary relationships among PaTCPs and the reliability of our phylogenetic analysis.

The TCP gene family is expanded (32 PaTCPs) in petunia compared with that in *Arabidopsis* (24 AtTCPs) but comparable to the family in tomato (30 SlTCPs), which may be the consequences of more genome duplication events in the Solanaceae family [50]. In addition, *PaTCP24a* and *PaTCP24b* were clustered into the same branch and located closely in the same assembled scaffold (Table 1, Figure 1), suggesting that tandem duplication may be another reason for the expansion of the *TCP* gene family in petunia. Interestingly, the number of protein-coding genes in *P. axillaris* genome (32,928) and *P. inflata* genome (36,697) are in strong correspondence with the fact that the number of protein-coding genes is 25,498 in the *Arabidopsis* genome [50]. From the phylogenetic tree, most *Arabidopsis* class II *TCP* genes contain more than one counterpart in petunia and tomato; especially in the CYC/TB1 subclade, where each gene has two or more counterparts (Figure 1), such as the *PaTCP1a/PaTCP1b* and *SlTCP7*/*SlTCP27* gene pairs vs. *AtTCP1*, *PaTCP12a*/*PaTCP12b* and *SlTCP22/SlTCP25* vs. *AtTCP12* and *PaTCP18a*/*PaTCP18b/PaTCP18c* and *SlTCP8/SlTCP9* vs. *AtTCP18*. Furthermore, there are two orthologs in petunia corresponding to one *SlTCP* in tomato and vice versa two tomato orthologs corresponding to one petunia gene for several members, such as *PaTCP24a* and *PaTCP24b* vs. *SlTCP24*, *PaTCP4a* and *PaTCP4b* vs. *SlTCP10*, *PaTCP19a* and *PaTCP19b* vs. *SlTCP20*, *SlTCP5* and *SlTCP28* vs. *PaTCP13*, as well as *SlTCP1* and *SlTCP30* vs. *PaTCP10* (Figure 1). It may be the consequence of the differential duplication events or frequencies of retaining copies after the divergence of tomato and petunia from a common ancestor. Only *AtTCP16* is separate without a close homolog in petunia, and a similar result was also observed in tomato [37], implying a lineage-specific gene loss in petunia and tomato.

### 3.2. Expression Patterns and Potential Functions of PaTCP Genes

The *TCP* gene family has become the focus of functional studies at multiple levels after its initial definition in 1999 [32]. At present, however, very little is known about the function of *TCP* genes in petunia. It is widely recognized that the expression profile of a gene is tightly linked with its function to a large extent. In this study, the expression profiles of *PaTCPs* in various tissues were investigated by qPCR. The results indicate that the transcript accumulation pattern in different tissues varies widely among *PaTCP* genes (Figure 5), suggesting their functional diversification during plant growth and development.

Previous studies have shown that the CIN subclade *TCP* genes mainly participate in the regulation of flowering time, floral organ development, leaf development and senescence, secondary cell wall thickening and morphogenesis of lateral organs [55,56]. In *Arabidopsis*, five *CIN*-like genes (*TCP2*, *3*, *4*, *10* and *24*) are post-transcriptionally regulated by microRNA319 (miR319). High levels of miR319 or low miR319-regulated *TCP* activity induce serrated and crinkled leaves due to excessive cell proliferation and delay leaf senescence by inhibiting the biosynthesis of jasmonic acid [8,10,33]. Proper regulation of *TCP4* by *miR319a* is critical for petal and stamen development [12]. In tomato, seven *SlTCP* genes are predicted to be the targets of miR319, and *LANCEOLATE* (*LA*, *SlTCP2*), regulated by miR319, is required for the formation of compound leaves [37,57]. In petunia, seven closest homologs of the *CIN*-like genes (*PaTCP3a*, *3b*, *4a*, *4b*, *10*, *24a* and *24b*) have the putative binding sites for PamiR319 (Figure 2c). High expression levels of *PaTCP3a*, *PaTCP4a/PaTCP4b*, *PaTCP10* and *PaTCP24a/PaTCP24b* in leaves suggest their potential roles in leaf development, which may be conserved for the *TCP* genes targeted by miR319 [8,55,57,58,59]. Moreover, the transcripts of most putative miR319-regulated *PaTCP* genes are detected in most tested tissues, including seedlings, cotyledons, leaves, axillary buds, inflorescences and flower buds, suggesting that these *PaTCPs* may play diverse regulatory roles at multiple developmental processes in petunia. Recently, the *TCP4*-like genes were proved to play a positive role in light-induced cotyledon opening via directly targeting and transcriptionally activating several *SAUR* (*SMALL AUXIN UPREGULATED RNA*) genes in *Arabidopsis* [60]. *AtTCP4* is predominantly expressed in cotyledons and young leaves [8] and regulates the morphogenesis of these organs [33]. Notably, *PaTCP4a*, as well as *PaTCP2*, shows the highest expression levels in the cotyledons, suggesting that they may have a similar role as *AtTCP4* in regulating cotyledon development and/or opening. In contrast, *PaTCP4b*, the duplicated sister gene of *PaTCP4a*, appears to have lost its expression in the cotyledons and seedlings, implying a possible subfunctionalization for *PaTCP4b* after gene duplication. This subfunctionalization or even neofunctionalization may also have occurred in *PaTCP1a/PaTCP1b*, *PaTCP12a/PaTCP12b* and *PaTCP1a/PaTCP1b* gene pairs based on their differential expression patterns (Figure 5). However, *PaTCP3a* and *PaTCP3b*, as well as *PaTCP24a* and *PaTCP24b*, show almost identical expression patterns, suggesting these gene pairs may be resulted from more recent duplications and have redundant functions.

*TCP5*, *TCP13* and *TCP17* (*TCP5*-like) are the *CIN*-like genes not regulated by miR319 in *Arabidopsis*. It has been suggested that *TCP5*/*13*/*17* play redundant regulatory roles in flowering time, leaf and petal growth and axillary branching [19,21,61,62]. Constitutive expression of *TCP5*, -*13* or -*17* accelerates flowering time [21], and *TCP5* overexpression also leads to smaller petals [61], while knockdown of all three genes by the T-DNA insertion or artificial miRNA, as well as the *tcp5/13/17* triple mutation, results in delayed flowering, with larger petals and leaves, although the single *tcp5, tcp13* or *tcp17* mutant shows no or weak phenotypic alterations [19,21,62]. In petunia, *PaTCP5*, *PaTCP13* and *PaTCP17* are the orthologs of *AtTCP5*, -*13* and -*17*, respectively. There is no target site of PamiR319 in these genes, indicating they are not regulated by miR319. Interestingly, *PaTCP5*, *PaTCP13* and *PaTCP17* all exhibit the highest level of expression in the inflorescences (Figure 5), implying that these genes probably perform conserved roles in controlling flower development and/or flowering time.

In core eudicots, the *CYC/TB1* subclade contains three main groups: *CYC1*, *CYC2* and *CYC3*. Increasing functional analyses in recent years indicate that the *CYC3*, and particularly *CYC1* genes, play a central and well-conserved regulatory role in the development of axillary meristems and shoot branching [63], whereas the *CYC2* genes are key players in the control of floral symmetry, inflorescence architecture and reproductive organ development [64]. Our study identified seven genes in petunia belonging to the members of *CYC/TB1* subclade, namely *PaTCP1a/b*, *PaTCP12a/b* and *PaTCP18a/b/c*, which are corresponding to the *CYC2*, *CYC3* and *CYC1* group, respectively. The three petunia *CYC1* group of genes (*PaTCP18a/b/c*) are all expressed predominantly in vegetative axillary buds (Figure 5), which is consistent with the expression patterns of *BRANCHED1*-like genes in other species, such as *AtBRC1* (*AtTCP18*) and *AtBRC2* (*AtTCP12*) in *Arabidopsis* [6], *PsBRC1* in pea [65], *SlBRC1s* in tomato [66] and *CsBRC1* in cucumber [67], suggesting that *PaTCP18a/b/c* have a conserved role in regulating axillary bud outgrowth and shoot branching. This was supported by the recent study that identified *PhTCP3* (corresponding to *PaTCP18c* in this study) acting downstream strigolactone (SL) signaling to regulate lateral branching in petunia [51]. In addition, downstream genes in SL signaling have been identified using a microarray in *Arabidopsis* [68]. It is interesting to further investigate the molecular mechanism for petunia *CYC1* group genes in SL pathway. The two *CYC3*-group genes, *PaTCP12a* and *PaTCP12b*, have a litter different expression pattern, with *PaTCP12a* mainly expressing in axillary buds followed by leaves and stems while *PaTCP12b* predominantly expressed in leaves, suggesting their potentially divergent roles in the axillary bud and leaf development. *AtTCP1*, the *CYC2* group member in *Arabidopsis*, exhibits strong expression in the dorsal (adaxial) region of the axillary shoot meristems and early-stage flower meristems, the petiole and midrib of leaves including cotyledons, the distal region of expanding rosette leaves, as well as the lower part of inflorescences stem, and play a role in the longitudinal elongation of leaves [26,69]. *PaTCP1a* and *PaTCP1b*, the two orthologs of *AtTCP1*, are transcribed at high levels in inflorescences that contain flower meristems and flower buds at an early stage of development, implying that these genes may participate in the regulation of inflorescence architecture and/or flower development. In addition, *PaTCP1b* is also highly expressed in roots, even higher than that in inflorescences, suggesting it has a potential function in root development. Taken together, the petunia *CYC/TB1*-like genes may have conserved roles in the control of lateral branching, inflorescence architecture and flower development, while some members may also have undergone functional divergence after gene duplication.

Class I *PaTCP* genes demonstrate two distinct expression patterns (Figure 5). Most genes, such as *PaTC6*, *PaTCP7*, *PaTCP11*, *PaTCP14a*, *PaTCP14b*, *PaTCP20* and *PaTCP23,* show a more widespread and less tissue-specific expression profile, which is consistent with the previous results described in *Arabidopsis* [36], watermelon [39] and grapevine [47], and also coincide with the diverse roles of Class I TCP genes in various biological processes and stress adaptation [56]. While several genes display a tissue-preferential expression pattern, such as *PaTC8*, *PaTCP9*, *PaTCP15* and *PaTCP22*. In *Arabidopsis*, the two best-characterized class I genes *AtTCP14* and *AtTCP15* are dynamically expressed in young proliferating tissues such as leaf primordia, young inflorescence stems, as well as young flower pedicels, and their expression gradually decreases as the tissues mature [70]. Functional analysis indicates *AtTCP14* and *AtTCP15* act redundantly to regulate internode elongation and plant stature by promoting cell proliferation in young stem internodes [70]. Furthermore, the *ClTCP14a* and *ClTCP15* of watermelon, closely related to *AtTCP14* and *AtTCP15*, were proven to associate with the internode elongation. Ectopic expression of either *ClTCP14a* or *ClTCP15* resulted in an increase in inflorescence height of wild-type *Arabidopsis* and was sufficient to restore the stem internode length and inflorescence height of *tcp14 tcp15* double-mutant [39]. In petunia, the transcripts of *PaTCP14a* and *PaTCP14b* (two orthologs of *AtTCP14*), as well as *PaTCP11*, *PaTCP15* and *PaTCP22*, were markedly detected in the stems and/or inflorescences, implying that these genes may have similar roles in the regulation of internode elongation of stems and/or inflorescences. In tomato, three class I genes *SlTCP12*, *SlTCP15* and *SlTCP18* are predominantly expressed in developing fruits and were suggested to participate in the regulation of tomato fruit ripening [37]. Noteworthily, *PaTCP21* and *PaTCP23* represent a relatively high level of expression in developing fruits, suggesting they may play roles in fruit development. *PaTCP9, PaTCP14b*, *PaTCP20*, *PaTCP21* and *PaTCP23* are transcribed at the highest levels in cotyledons, suggesting their potential roles in cotyledon development, similar to the *TCP4*-like genes in *Arabidopsis* [8,33,60].

### 3.3. Potential Roles of PaTCP Genes during Petal Development

The TCP family has been reported to play key roles in the development of lateral organs, which mainly relies on direct transcriptional regulation of cell cycle genes and several hormone pathways [17]. In this study, we investigated the transcriptional profiles of *PaTCPs* during petal development in large- and small-flowered lines, respectively (Figure 7, Figure S3). As a result, most CIN-TCP genes such as *PaTCP3a*, *PaTCP4a/b*, *PaTCP13*, *PaTCP17* and *PaTCP24a/b* were shown to be expressed at relatively high levels in developing petals of both large- and small-flowed lines (Figure 7), which is consistent with their high levels of expression in flower buds relative to the CYC-TCP members (Figure 5). In addition, these CIN-TCP genes mostly showed gradually increasing expression during the early petal development stages or throughout the whole petal development process, suggesting they function in petal development in petunia. In most species, *CIN-TCPs* show dynamic expression patterns in the leaves that are undergoing morphogenesis, which is spatiotemporally related to the arrest front during leaf development, indicating they function as repressors of cell division [55]. The upregulation of most petunia *CIN-TCPs* during petal development is consistent with their role as growth repressors to inhibit cell proliferation and promote post-mitotic differentiation at later stages of fetal development. In *Arabidopsis*, *RABBIT EARS* (*RBE*) inhibits the expression of *TCP4* and *TCP5* to promote cell proliferation and petal growth during early petal development. However, this repression is alleviated at late developmental stages, which is required for the proper transition from cell division to cell expansion [19,71]. However, it is worth mentioning that *PaTCP3b* and *PaTCP4a* showed significantly higher levels of expression in the petals of large flowers (‘L’) than that of small flowers (‘S’), implying that they may play a role in promoting petal growth, which is contradictory with the function of *Arabidopsis TCP4* and *TCP5* in petal growth, but similar to the role of *CIN*, a *TCP4* homolog in *Antirrhinum majus*. *CIN* represses cell proliferation and promotes growth arrest in leaves, but it promotes growth in petals [72]. It is necessary to characterize the functions of petunia *CIN-TCP* genes, especially *PaTCP3b* and *PaTCP4a,* by further studies.

For class I *TCPs*, most *PaTCPs* showed very low levels of expression in flower buds and petals, except for *PaTCP8, PaTCP14a, PaTCP19a* and *PaTCP22*, which have relatively high expression (more than 0.005 relatives to *PhEF1α*) during petal development (Figure 5 and Figure 7). In contrast to the *CIN-TCPs*, these *PaTCPs* mostly showed decreasing expression trends during the petal growth process and have higher expression levels in ‘S’ than ‘L,’ suggesting they play opposite roles to the *CIN-TCPs* in petal development. Previously, class I TCP genes are suggested to promote cell proliferation and concomitant organ growth. For example, *AtTCP20* is involved in stimulation of cell growth and division [73]. Overexpressing *CmTCP20* in *Arabidopsis* and chrysanthemum plants exhibit larger inflorescences and flower buds and longer petal length [74]. Again, the expression patterns of *PaTCP14a/b* and *PaTCP19b* during the petal development support their functions to promote cell proliferation, while higher expression in the petals of small flowers is contrary to the promoting role in petal growth, which needs further study to be understood.

Our promoter analysis shows that most *PaTCP* genes harbor plenty of hormone-related *cis*-regulatory elements (Figure 6), implying that these genes may have profound effects on plant developmental processes downstream of hormone pathways. Previous genetic studies indicate that phytohormones and transcription factors have vital roles in different pathways responsible for the determination of flower size in *Arabidopsis* [75]. Cytokinin is reported to affect the duration of cell division in *Arabidopsis* developing floral organs [76]. Similarly, it has been reported that cytokinin application accelerates the cell proliferation rate and prolongs the duration of cell proliferation phase during corolla development in petunia, resulting in the formation of larger flowers [77]. The extending corollas of the large- and small-flowered lines ‘L’ and ‘S’ were harvested to measure the levels of auxin, cytokinin, brassinosteroids, ethylene and JA. The levels of IAA, BL, ACC and JA in the corollas of ‘S’ were significantly higher than those of ‘L,’ and the cytokinin level in ‘S’ was also higher than that in ‘L’ (unpublished), which is consistent with previous results [78]. Considering the distribution of a plentiful of hormone-responsive elements in the promoter regions of *PaTCP* genes (Figure 6), together with accumulating functional studies of the effects of TCPs on hormone biosynthesis, transport and signal transduction in tissues and organs, it could be postulated that *PaTCP* genes might function as key mediators of hormone-induced changes in plant growth and development. These results indicate that these phytohormone levels are tightly linked with petunia petal size. Taken together, PaTCP transcription factors and phytohormone levels may have an interactional and coefficient relationship in the development of floral organ size in petunia.

## 4. Materials and Methods

### 4.1. Plant Materials

*Petunia axillaris* (S26) and two petunia selfing lines ‘L’ and ‘S,’ with large and small flowers, respectively, were used in this study. The seeds of S26 were originally obtained from the Swammerdam Institute for Life Sciences, University of Amsterdam, The Netherlands (gifted by prof. Ronald Koes), and the two selfing lines were developed by our laboratory. Plant materials were grown in phytotron under a long-day condition (16 h light/8 h dark) at 22–25 °C with a luminous intensity of 12,000 lux and relative humidity of 70–80%.

### 4.2. Identification of TCP Genes from Petunia Species

Two different approaches were used to identify TCP genes in petunia genomes. Firstly, petunia TCP genes were retrieved from *P. axillaris* N and *P. inflata* S6 genome databases [50] in the Sol Genomics Network (SGN) (https://solgenomics.net/) using the TBLASTN algorithm with the hidden Markov model (HMM) for the TCP domain (PF03634) obtained from the Pfam database (http://pfam.sanger.ac.uk). Secondly, the TCP protein sequences of 24 *Arabidopsis* TCPs and 30 tomato TCPs, which were downloaded from the Arabidopsis Information Resource (TAIR) database (http://www.arabidopsis.org) and the Solanaceae Genomics Network (https://solgenomics.net/), respectively, were used as queries to search the genome datasets of *P. axillaris* and *P. inflata* [50]. Furthermore, all obtained TCP sequences from petunia were put in InterproScan (http://www.ebi.ac.uk/InterProScan) to confirm the presence of the TCP domain. Subsequently, the TSA (Transcriptome Shotgun Assembly) databases of *P. axillaris* (GBRU) and *P. integrifolia* (GBRV) as well as *P. integrifolia* subsp. *inflata* (GBDS) were searched for the transcripts of the identified *PaTCPs* and *PiTCPs* genes by nucleotide BLAST in the NCBI [52]. The molecular weight (MW) and isoelectric points (pI) of PaTCP and PiTCP proteins were computed by the ExPASy website (https://web.expasy.org/protparam/).

### 4.3. Multiple Sequence Alignments and Phylogenetic Analysis

TCP protein sequences of the 24 AtTCPs and 32 SlTCPs were retrieved from TAIR (http://www.arabidopsis.org/) and the tomato genome database in SGN, respectively. Multiple sequence alignments of the full-length protein sequences of TCP proteins of petunia, *Arabidopsis* and tomato were conducted using the Clustal W program of MEGA 7.0 software. An unrooted phylogenetic tree based on the alignments was constructed by the neighbor-joining (NJ) method using Jones-Taylor-Thornton (JTT) substitution model with Gamma-distributed rates (five categories) and bootstrap values were calculated with 500 replicates.

### 4.4. Gene Structure and Conserved Motif Analysis

The exon-intron structure of 66 petunia genes (32 *PaTCPs* from *P. axillaris* and 34 *PiTCPs* from *P. inflata*) was analyzed using the Gene Structure Display Server 2.0 (http://gsds.cbi.pku.edu.cn/).

The conserved TCP domains of PaTCP proteins were confirmed by the Conserved Domain Database (CDD) in NCBI with default parameters. The conserved and potential motifs of 32 PaTCP protein sequences were analyzed by the online Multiple Expectation Maximization for Motif Elicitation (MEME) (http://meme-suite.org/tools/meme) with the optimized parameter settings: repetition number, any; minimum motif width, 6; maximum motif width, 50; maximum number of motifs, 20.

### 4.5. PamiR319-Targeted Site Prediction

The mature sequences of PamiR319 were obtained from the miRbase Database (http://www.mirbase.org). The coding sequences of 32 *PaTCP* genes and mature sequences of PamiR319 (Table S4) were analyzed using the psRNATarget online application (http://plantgrn.noble.org/psRNATarget/) with default parameters.

### 4.6. Putative Cis-Element Analysis of PaTCP Promoters

The promoter sequences of 32 *PaTCPs* genes were retrieved from *P. axillaris* genome database and the promoter sequences of upstream 1.5 kb region from the start codon for each gene were used for putative promoter *cis*-acting element analysis in PlantCARE online program (http://bioinformatics.psb.ugent.be/webtools/plantcare/html/).

### 4.7. RNA Isolation and Real-Time Quantitative RT-PCR Analysis

For the investigation of spatial expression profiles of *PaTCP* genes, different vegetative and reproductive tissues were harvested from *P. axillaris*, including germinating seeds (3 d (day) after sowing), cotyledons (7 d after sowing), 4-euphylla seedlings, roots, stems, leaves, axillary buds of adult plants (after flowering), inflorescences, flower buds (0.5 cm) and immature fruits (7 d after pollination). Moreover, samples used in the analysis of the temporal expression patterns of *PaTCP* genes were taken from the large- and small-flowered lines of ‘L’ and ‘S’ at five different floral developmental stages, including young flower buds (petal length < 0.5 cm, S1), extending flower buds (when flower buds just enclosed by sepals, S2), pre-anthesis flowers (when flower buds extended to full length, S3), semi-open flowers (S4) and fully blooming flowers before the anthers dehisced (S5), respectively. Corollas were detached from flower buds at each development stages, with the sepals, stamens and pistils removed, and then frozen in liquid nitrogen immediately.

RNA extraction and qPCR were performed as described previously [79]. Gene-specific primers for *PaTCPs* were designed using Primer 5.0 and were listed in Table S4. The amplification efficiency of each primer pair was examined by melting curve analysis. For all qPCR experiments, three biological replicates were analyzed for each sample and the mean values ± SD (standard deviation) were calculated. The petunia *EF1α* gene was used as the internal control. The 2 ^−ΔΔCT^ method [80] was used for calculating the relative expression level of each gene.

### 4.8. Subcellular Localization of PaTCP Proteins

The full-length coding sequences of *PaTCP3a*, *PaTCP4a* and *PaTCP12b*, except for the stop codon, were amplified (primers are listed in Table S4) and inserted into the pMD18-T vector (Takara, Japan). Digestion of these vectors was conducted using KpnI and Sal I (Thermo Fisher Scientific, Waltham mass, MA, USA) restriction endonuclease enzymes. Then, the digested products were purified and immediately ligated into the appropriate sites of the 35S promoter-driven yellow fluorescent protein (YFP)-fusion vector (L101-YFP). All the constructed plasmids were confirmed by sequencing. The *Agrobacterium tumefaciens* EHA105 strains harboring fusion constructs were transformed into tobacco epidermal cells. Signals were detected by confocal laser microscopy (Leica SP8).

## 5. Conclusions

In this work, 32 *PaTCP* genes in *P. axillaris* and 34 *PiTCP* genes in *P. inflata* were identified, respectively. For *PaTCP* genes, we carried out a comprehensive analysis and characterization. Based on the multiple sequence alignments and phylogenetic analysis, 32 *PaTCP* genes were categorized into two classes, which was supported by the highly conserved exon-intron structure and motif compositions. The spatial-temporal expression analysis revealed that the *PaTCP* genes may be involved in multiple aspects of plant growth and development. Remarkably, some *PaTCP* genes showed significantly different expression levels between the small-flowered line ‘S’ and the large-flowered line ‘L,’ suggesting their potential roles in petal development and/or specification of petal size. Moreover, the number of *cis*-regulatory elements observed in the *PaTCP* promoter sequences are likely to be conducive to mediate hormone and/or stress responses. Taken together, the extensive genome-wide analysis of *PaTCP* genes lays a solid foundation for unraveling the potential functions of *PaTCPs* during plant growth and development.

## Figures and Tables

**Table 1:** *TCP* genes identified in *Petunia axillaris.*

Gene Name	GenBank Accession Number	Genomic Location	Protein Length (aa)	*M*_W_ (Da)	pI	Type
*PaTCP2*	MT293300	Peaxi162Scf00939: 373844-373149	231	25,346.90	4.49	CIN
*PaTCP3a*	MT293301	Peaxi162Scf00069: 1992329-1991073	418	45,694.00	6.27	CIN
*PaTCP3b*	MT293302	Peaxi162Scf00000: 2512603-2513853	416	45,483.18	6.11	CIN
*PaTCP4a*	MT293303	Peaxi162Scf00189: 194164-193223	313	35,529.20	6.59	CIN
*PaTCP4b*	MT293304	Peaxi162Scf00241: 1053171-1054422	314	35,195.08	6.46	CIN
*PaTCP5*	MT293305	Peaxi162Scf00013: 698884-699834	316	35,316.19	8.67	CIN
*PaTCP10*	MT293310	Peaxi162Scf00269: 345789-344431	452	50,862.17	5.87	CIN
*PaTCP13*	MT293314	Peaxi162Scf00166: 407926-406976	316	35,253.97	7.95	CIN
*PaTCP17*	MT293318	Peaxi162Scf00201: 332740-333747	335	37,477.28	5.61	CIN
*PaTCP24a*	MT293328	Peaxi162Scf00317: 44365-45591	408	45,311.45	5.83	CIN
*PaTCP24b*	MT293329	Peaxi162Scf00317: 35045-36322	425	46,728.30	6.61	CIN
*PaTCP1a*	MT293298	Peaxi162Scf00479: 76745-78241	362	40,884.15	9.75	CYC
*PaTCP1b*	MT293299	Peaxi162Scf00535: 12436-13766	333	37,208.83	9.65	CYC
*PaTCP12a*	MT293312	Peaxi162Scf00086: 565892-567112	406	46,671.52	6.48	CYC
*PaTCP12b*	MT293313	Peaxi162Scf00367: 862637-863788	383	43,907.64	6.33	CYC
*PaTCP18a*	MT293319	Peaxi162Scf00119: 242135-243400	343	39,094.66	9.07	CYC
*PaTCP18b*	MT293320	Peaxi162Scf00013: 930102-931799	365	41,213.33	5.97	CYC
*PaTCP18c*	MT293321	Peaxi162Scf00015: 735479-737065	341	38,603.76	7.25	CYC
*PaTCP6*	MT293306	Peaxi162Scf00128: 1570847-1571590	247	27,160.02	8.76	PCF
*PaTCP7*	MT293307	Peaxi162Scf00681: 139168-139935	255	26,952.13	9.24	PCF
*PaTCP8*	MT293308	Peaxi162Scf00011: 881162-882733	523	56,100.68	6.82	PCF
*PaTCP9*	MT293309	Peaxi162Scf00021: 216329-217791	309	33,695.65	10.01	PCF
*PaTCP11*	MT293311	Peaxi162Scf00123: 1704660-1705283	207	22,320.35	8.86	PCF
*PaTCP14a*	MT293315	Peaxi162Scf00013: 171612-173102	332	36,667.84	9.44	PCF
*PaTCP14b*	MT293316	Peaxi162Scf00274: 795778-797097	413	44,322.68	6.86	PCF
*PaTCP15*	MT293317	Peaxi162Scf00541: 598300-599367	355	38,885.66	7.02	PCF
*PaTCP19a*	MT293322	Peaxi162Scf00192: 477569-478627	352	37,603.70	5.12	PCF
*PaTCP19b*	MT293323	Peaxi162Scf00620: 802496-803548	350	37,896.90	9.23	PCF
*PaTCP20*	MT293324	Peaxi162Scf00420: 631358-632224	288	31,161.43	7.94	PCF
*PaTCP21*	MT293325	Peaxi162Scf00021: 2123474-2124109	211	22,362.10	9.46	PCF
*PaTCP22*	MT293326	Peaxi162Scf00391: 454319-455563	414	44,056.39	7.40	PCF
*PaTCP23*	MT293327	Peaxi162Scf00268: 644156-645127	323	34,471.24	7.89	PCF

aa: Amino acid; pI: Isoelectric point; *M*_W_: Molecular weight.

## Supplementary Materials

The following are available online at https://www.mdpi.com/1422-0067/21/18/6594/s1.

## Abbreviations

ABA	Abscisic acid
ACC	1-aminocyclopropane-1-carboxylic acid
bHLH	Basic helix-loop-helix
BR	Brassinosteroids
CYC	Cycloidea
IAA	Indole-3-acetic acid
JA	Jasmonic acid
MeJA	Methyl jasmonate
MW	Molecular weight
NJ	Neighbor-Joining
PCF	Proliferating cell factor
pI	Isoelectric point
qPCR	Real-time quantitative RT-PCR
SL	Strigolactones
TB1	Teosinte branched1
TFs	Transcription factors
UTR	Untranslated region
YFP	Yellow fluorescence protein
