# Supplementary material for: Genome-Wide Identification, Characterization and Expression Analysis of TCP Transcription Factors in Petunia
Source: Int J Mol Sci. 2020 Sep 9;21(18):6594. doi: 10.3390/ijms21186594 (PMC7554992; doi:10.3390/ijms21186594)
Supplement: Supplementary file 1 [file ijms-21-06594-s001.zip › ijms-910540-supplementary/IJMS_PDF/Figure S1.pdf]

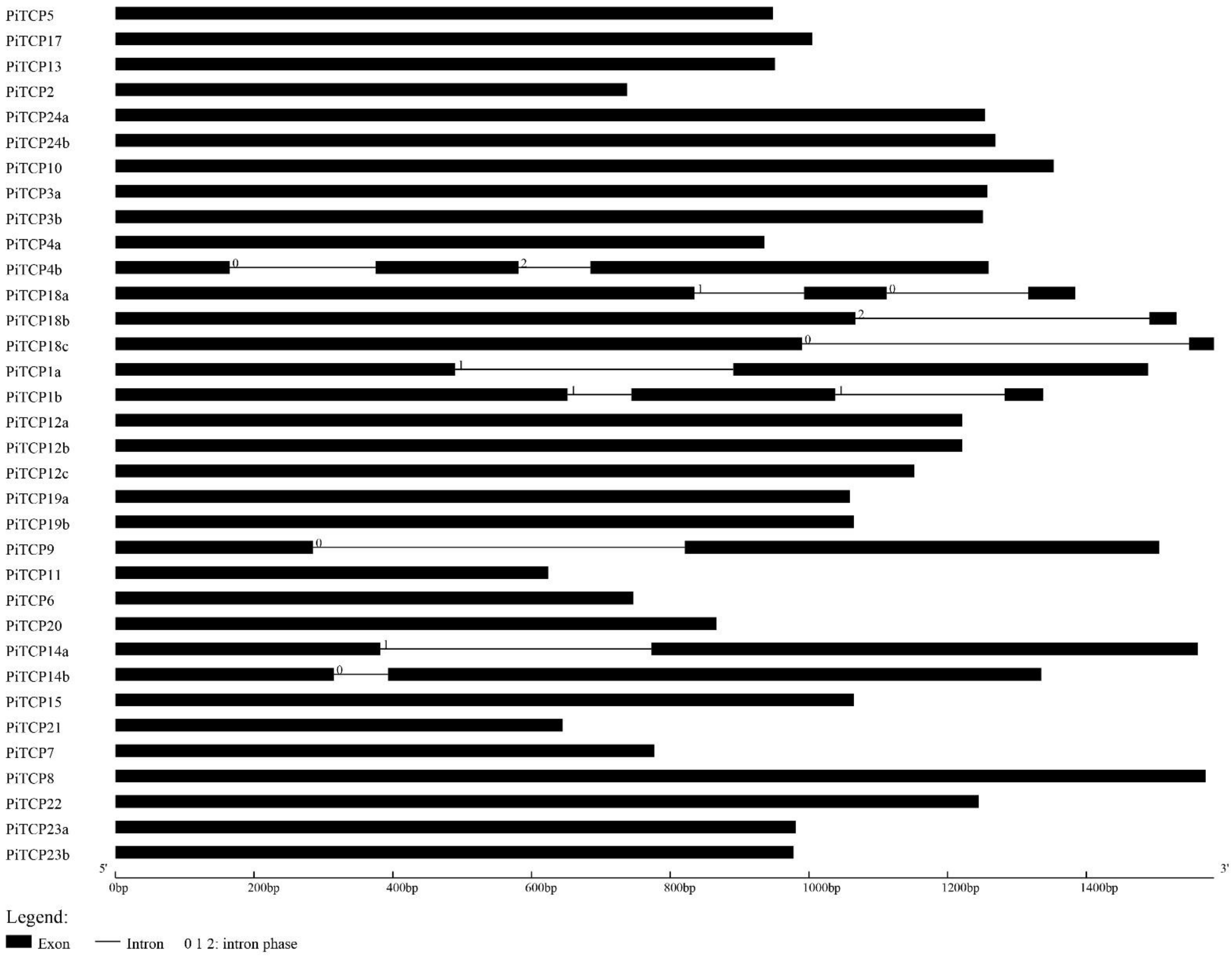

Figure S1. Exon-intron structures of *PiTCP* genes. 5'UTR and 3'UTR were not showed. The exons and introns were indicated by black block and gray thin line, respectively. The 0, 1 and 2 represented intron phases.
