# Supplementary material for: Genome-Wide Identification, Characterization and Expression Analysis of TCP Transcription Factors in Petunia
Source: Int J Mol Sci. 2020 Sep 9;21(18):6594. doi: 10.3390/ijms21186594 (PMC7554992; doi:10.3390/ijms21186594)
Supplement: Supplementary file 1 [file ijms-21-06594-s001.zip › ijms-910540-supplementary/IJMS_PDF/Figure S2.pdf]

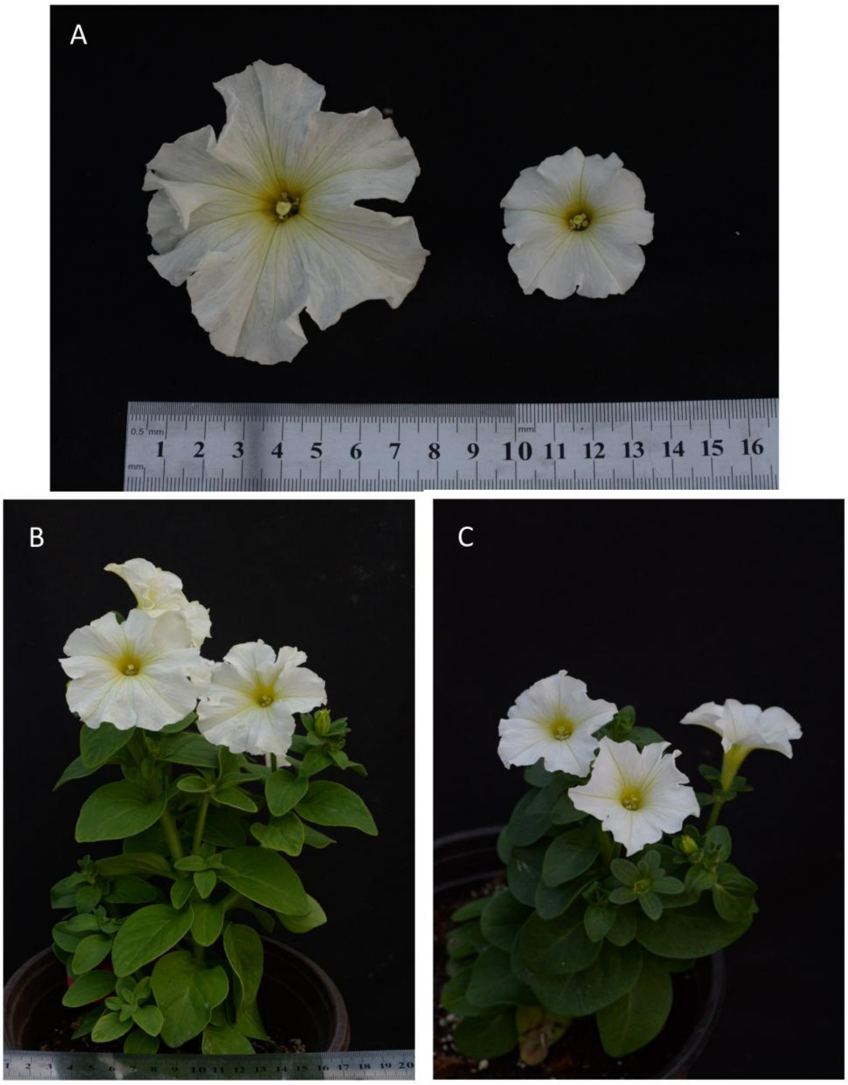

Figure S2. Photos of the large- and small-flowered lines ‘L’ and ‘S’. (A) Presentation of flower diameters of ‘L’ and ‘S’, ‘L’ (left) and ‘S’(right). (B) ‘L’ and (C) ‘S’.
