# Supplementary material for: Genome-Wide Identification, Characterization and Expression Analysis of TCP Transcription Factors in Petunia
Source: Int J Mol Sci. 2020 Sep 9;21(18):6594. doi: 10.3390/ijms21186594 (PMC7554992; doi:10.3390/ijms21186594)
Supplement: Supplementary file 1 [file ijms-21-06594-s001.zip › ijms-910540-supplementary/IJMS_PDF/Figure S3.pdf]

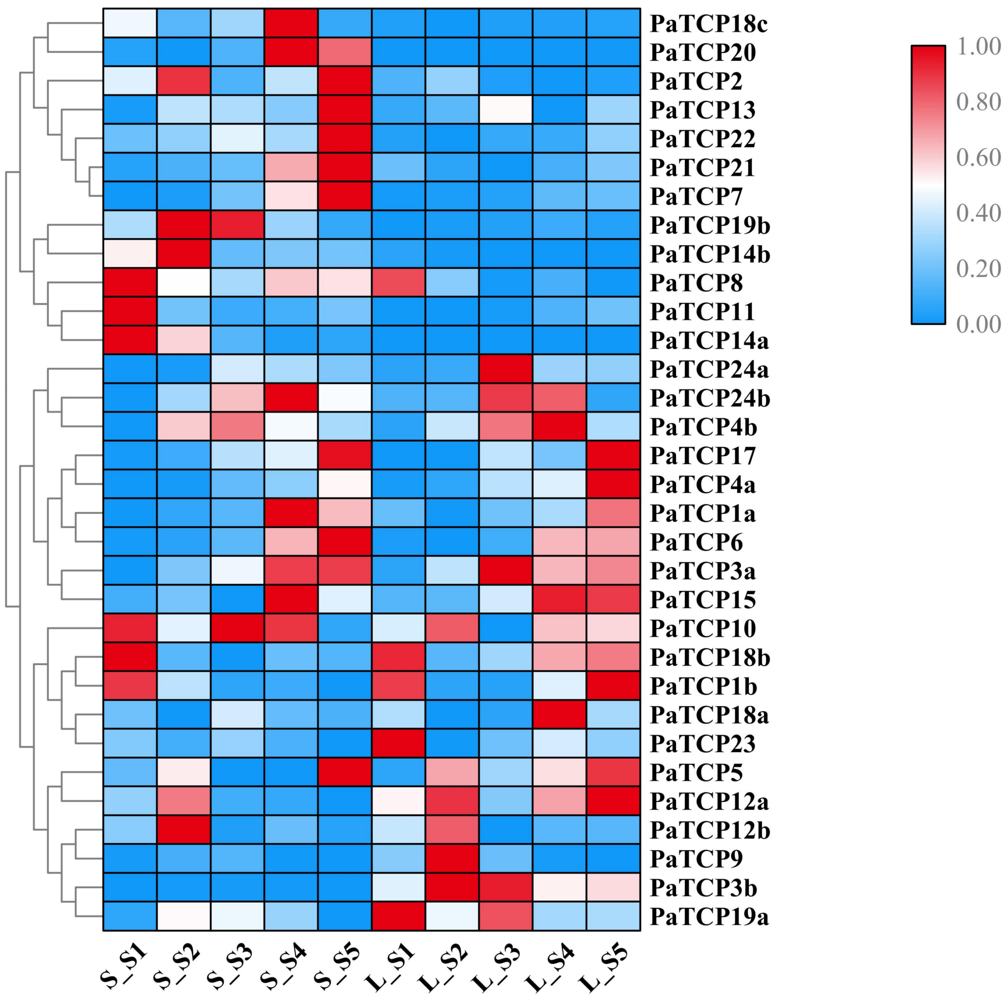

Figure S3. Heat map representation for the expression profiles of *PaTCP* genes in the large- and small-flowered lines 'L' and 'S' during different petal developmental stages. The expression levels are shown in color bar according to the scale. Samples were designated as S_S1 to S_S5 and L_S1 to L_S5, which represented the 'S' and 'L' at five developmental stages, respectively. S1, young flower buds (< 0.5 cm,); S2, extending flower buds (when flower buds just enclosed by sepals); S3, pre-anthesis (when flower buds extended to full length); S4, semi-open flowers; S5, fully blooming flowers before the anthers dehisced.
