# Supplementary material for: Genome-Wide Identification, Characterization and Expression Analysis of TCP Transcription Factors in Petunia
Source: Int J Mol Sci. 2020 Sep 9;21(18):6594. doi: 10.3390/ijms21186594 (PMC7554992; doi:10.3390/ijms21186594)
Supplement: Supplementary file 1 [file ijms-21-06594-s001.zip › ijms-910540-supplementary/IJMS_PDF/Table S1.pdf]

Table S1. Additional information of *PaTCP* genes in *P. axillaris*.

Gene name	Scaffold ID [a]	Regions of exons (bp)	Length of gDNA (bp)	Length of ORFs (bp)	Right transcripts in TSA database[b]
PaTCP2	Peaxi162Scf00939	373844-373149 (696)	696	696	GBRU01035768.1
PaTCP3a	Peaxi162Scf00069	1992329-1991073 (1257)	1257	1257	GBRU01005986.1
PaTCP3b	Peaxi162Scf00000	2512603-2513853 (1251)	1251	1251	GBRU01039650.1
PaTCP4a	Peaxi162Scf00189	194164-193223 (942)	942	942	GBRU01063454.1
PaTCP4b	Peaxi162Scf00241	1053171-1053335 (165);<br>1053551-1053756 (206);<br>1053849-1054422 (574)	1252	945	GBRU01039883.1
PaTCP5	Peaxi162Scf00013	698884-699834 (951)	951	951	GBRU01068616.1
PaTCP10	Peaxi162Scf00269	345789-344431 (1359)	1359	1359	/
PaTCP13	Peaxi162Scf00166	407926-406976 (951)	951	951	GBRU01068599.1
PaTCP17	Peaxi162Scf00201	332740-333747 (1008)	1008	1008	GBRU01068889.1
PaTCP24a	Peaxi162Scf00317	44365-45591 (1227)	1227	1227	GBRU01035325.1
PaTCP24b	Peaxi162Scf00317	35045-36322 (1278)	1278	1278	GBRU01044212.1
PaTCP1a	Peaxi162Scf00479	76745-77234 (490);<br>77643-78241 (599)	1497	1089	/
PaTCP1b	Peaxi162Scf00535	13766-13115 (652);<br>13022-12729 (294);<br>12493-12436 (56)	1331	1002	/
PaTCP12a	Peaxi162Scf00086	567112-565892 (1221)	1221	1221	GBRU01066955.1
PaTCP12b	Peaxi162Scf00367	863788-862637 (1152)	1152	1152	GBRU01068756.1
PaTCP18a	Peaxi162Scf00119	243400-242569 (832);<br>242481-242312 (170);<br>242164-242135 (30)	1266	1032	GBRU01074139.1 (Partial)
PaTCP18b	Peaxi162Scf00013	930102-931160 (1059);<br>931761-931799 (39)	1698	1098	/
PaTCP18c	Peaxi162Scf00015	737065-736076 (990);<br>735514-735479 (36)	1587	1026	GBRU01007607.1
PaTCP6	Peaxi162Scf00128	1570847-1571590 (744)	744	744	GBRU01050764.1
PaTCP7	Peaxi162Scf00681	139935-139168 (768)	768	768	GBRU01019482.1

PaTCP8	Peaxi162Scf00011	881162-882733 (1572)	1572	1572	GBRU01046126.1
PaTCP9	Peaxi162Scf00021	217791-217516 (276);<br>216982-216329 (654)	1463	930	GBRU01068661.1<br>(Partial)
PaTCP11	Peaxi162Scf00123	1705283-1704660 (624)	624	624	GBRU01036689.1
PaTCP14a	Peaxi162Scf00013	173102-172724 (379);<br>172415-171888 (528);<br>171703-171612 (92)	1491	999	GBRU01065321.1
PaTCP14b	Peaxi162Scf00274	795778-796095 (318);<br>796174-797097 (924)	1320	1242	GBRU01066596.1
PaTCP15	Peaxi162Scf00541	598300-599367 (1068)	1068	1068	GBRU01066056.1<br>（Partial）
PaTCP19a	Peaxi162Scf00192	477569-478627 (1059)	1059	1059	GBRU01004120.1
PaTCP19b	Peaxi162Scf00620	802496-803548 (1053)	1053	1053	GBRU01067781.1<br>(Partial)
PaTCP20	Peaxi162Scf00420	631358-632224 (867)	867	867	GBRU01046437.1
PaTCP21	Peaxi162Scf00021	2124109-2123474 (636)	636	636	GBRU01014247.1
PaTCP22	Peaxi162Scf00391	455563-454319 (1245)	1245	1245	GBRU01011275.1
PaTCP23	Peaxi162Scf00268	645127-644156 (972)	972	972	GBRU01067971.1

[a] Scaffold ID corresponds to the annotations provided by https://solgenomics.net/organism/Petunia_axillaris/genome (v1.6.2).

[b] The transcripts are retrieved from the TSA (Transcriptome Shotgun Assembly) database of *P. axillaris* (GBRU) in NCBI by nucleotide BLAST search.

'/' indicates no orthologous transcript was found. Partial indicates that the ORF sequences were incomplete.
