# Supplementary material for: Genome-Wide Identification, Characterization and Expression Analysis of TCP Transcription Factors in Petunia
Source: Int J Mol Sci. 2020 Sep 9;21(18):6594. doi: 10.3390/ijms21186594 (PMC7554992; doi:10.3390/ijms21186594)
Supplement: Supplementary file 1 [file ijms-21-06594-s001.zip › ijms-910540-supplementary/IJMS_PDF/Table S2.pdf]

Table S2. *PiTCP* genes in *P. inflata*.

Gene name	Scaffold ID[a]	Regions of exons (bp)	Gene length of gDNA (bp)	Length of ORFs (bp)	Protein length (aa)	MW(Da)	pI	Type	Right transcripts in TSA database[b]
PiTCP2	Peinf101Scf00576	317477-318214 (738)	738	738	245	26918.85	4.80	CIN	/
PiTCP3a	Peinf101Scf00665	44699-45955 (1257)	1257	1257	418	45566.88	6.37	CIN	GBRV01061558.1
PiTCP3b	Peinf101Scf00214	914017-915267 (1251)	1251	1251	416	45451.21	6.00	CIN	GBRV01097507.1
PiTCP4a	Peinf101Scf01267	324299-325234 (936)	936	936	311	35241.86	6.48	CIN	GBRV01057518.1
PiTCP4b	Peinf101Scf00830	378525-378689 (165); 378900-379105 (206); 379210-379783 (574)	1259	945	314	35192.08	6.46	CIN	GBRV01069684.1
PiTCP5	Peinf101Scf00500	380639-381586 (948)	948	948	315	35002.88	8.67	CIN	GBRV01099955.1+ GBDS01050375.1
PiTCP10	Peinf101Scf00058	228866-230218 (1353)	1353	1353	450	50647.94	5.77	CIN	GBRV01098730.1 (Partial)
PiTCP13	Peinf101Scf00359	21898-22848 (951)	951	951	316	35178.86	7.27	CIN	GBRV01101937.1
PiTCP17	Peinf101Scf01556	348320-349324 (1005)	1005	1005	334	37407.16	5.99	CIN	GBRV01099083.1
PiTCP24a	Peinf101Scf01317	1026352-1025099 (1254)	1254	1254	417	46321.46	5.97	CIN	GBDS01002613.1
PiTCP24b	Peinf101Scf01317	1038798-1037530 (1269)	1269	1269	422	46603.19	6.43	CIN	GBRV01064915.1+ GBRV01086822.1
PiTCP1a	Peinf101Scf00475	406521-406032 (490); 405630-405032 (599)	1490	1089	362	40779.05	9.73	CYC	/

PiTCP1b	Peinf101Scf00359	909385-908734 (652);<br>908641-908348 (294);<br>908103-908048 (56)	1338	1002	333	37247.81	9.55	CYC	/
PiTCP12a	Peinf101Scf01705	68828-70048 (1221)	1221	1221	406	46543.41	6.40	CYC	GBRV01101606.1
PiTCP12b	Peinf101Scf01034	334489-335709 (1221)	1221	1221	406	46543.41	6.40	CYC	/
PiTCP12c	Peinf101Scf00441	117555-116404 (1152)	1152	1152	383	44218.08	6.48	CYC	GBRV01098645.1
PiTCP18a	Peinf101Scf01642	312243-311409 (836);<br>311250-311134 (117);<br>310927-310860 (68)	1384	1020	339	38768.40	9.22	CYC	GBRV01026228.1
PiTCP18b	Peinf101Scf00500	166075-167142 (1068);<br>167567-167605 (39)	1531	1107	368	42112.70	6.48	CYC	/
PiTCP18c	Peinf101Ctg13694<br>859	516-1505 (990);<br>2064-2099 (36)	1584	1026	341	38603.76	7.25	CYC	GBRV01046363.1
PiTCP6	Peinf101Scf00889	317919-318665 (747)	747	747	248	27278.11	8.76	PCF	GBRV01003182.1
PiTCP7	Peinf101Scf00832	377626-378402 (777)	777	777	258	27386.63	9.24	PCF	GBRV01072957.1
PiTCP8	Peinf101Scf03443	195735-197306 (1572)	1572	1572	523	55993.52	6.82	PCF	GBRV01031545.1
PiTCP9	Peinf101Scf00175	1031106-1031390 (285);<br>1031927-1032610 (684)	1505	969	322	35099.27	10.06	PCF	/
PiTCP11	Peinf101Scf00871	606347-606970 (624)	624	624	207	22360.40	8.86	PCF	GBRV01054059.1
PiTCP14a	Peinf101Scf00500	891467-891848 (382);<br>892240-893027 (788)	1561	1170	389	42542.72	6.93	PCF	GBDS01011129.1+<br>GBRV01101117.1<br>(Partial)

PiTCP14b	Peinf101Scf01142	1103767-1103453 (315); 1103374-1102433 (942)	1335	1257	418	44873.29	7.06	PCF	GBRV01101229.1
PiTCP15	Peinf101Scf00077	838601-839665 (1065)	1065	1065	354	38805.66	7.36	PCF	GBRV01102148.1
PiTCP19a	Peinf101Scf01452	568938-569996 (1059)	1059	1059	352	37645.78	5.12	PCF	GBRV01046489.1
PiTCP19b	Peinf101Scf01166	879409-880473 (1065)	1065	1065	354	38340.31	8.66	PCF	GBDS01006482.1
PiTCP20	Peinf101Scf01192	419921-420787 (867)	867	867	288	31197.42	6.50	PCF	GBRV01045623.1
PiTCP21	Peinf101Scf01438	33393-34037 (645)	645	645	214	22513.27	9.46	PCF	GBRV01022696.1
PiTCP22	Peinf101Scf02974	481059-482303 (1245)	1245	1245	414	44085.47	7.40	PCF	GBDS01002716.1
PiTCP23a	Peinf101Scf02715	45288-46268 (981)	981	981	326	34636.40	8.63	PCF	GBRV01081713.1
PiTCP23b	Peinf101Scf01245	374903-375880 (978)	978	978	325	34541.23	8.63	PCF	GBRV01081712.1

[a] Sequence ID corresponds to the annotations provided by https://solgenomics.net/organism/Petunia_inflata/genome (v1.0.1).

[b] The transcripts were retrieved from the TSA (Transcriptome Shotgun Assembly) database of *P. integrifolia* (GBRV) and *P. integrifolia* subsp. *inflata* (GBDS) in the NCBI by nucleotide BLAST search.

'/' indicates no orthologous transcript was found. Partial indicated that the ORF sequences were incomplete.

aa, amino acid; MW, molecular weight; pI, isoelectric point.
