# Supplementary material for: Genome-Wide Identification, Characterization and Expression Analysis of TCP Transcription Factors in Petunia
Source: Int J Mol Sci. 2020 Sep 9;21(18):6594. doi: 10.3390/ijms21186594 (PMC7554992; doi:10.3390/ijms21186594)
Supplement: Supplementary file 1 [file ijms-21-06594-s001.zip › ijms-910540-supplementary/IJMS_PDF/Table S4.pdf]

Table S4. Primers used in this study and mature sequences of *PamiR319*.

A. Sequences of the primers for qRT-PCR

Gene name	The forward primer sequence (5′-3′)	The reverse primer sequence (5′-3′)
PaTCP1a	CAAGCCAGTGAAGGACCAAACA	AGCCAAAGTTTACCTTGCGATG
PaTCP1b	TTGCTGTTGTAACTTTCGTGCG	GGAAAGCCCAGTCCACACATTA
PaTCP2	ACAGGGTGGGGAGGTGACAG	ATTGTTTTGGGGTGATTGGCAG
PaTCP3a	GCTCAGCATCATAATAACCCCAA	TCCTGTGACTGCCCCACTACCA
PaTCP3b	ACACACTGGCATCGCTCTTGGT	GGATAGCAATCGCTGATGGGTCT
PaTCP4a	ATGACCAAGCAGCCCTTTTCTCTA	CGGAACTCTGTGGTGAAATGTCTG
PaTCP4b	CGGAAGCAACTTATCCAAGAATGA	CGAGAATAGGGAGAGCCAGAAGA
PaTCP5	GAGCCAATCGAATCTACGTGTCAT	CGCTTTAGTTTCTTGAGCAGTCCAG
PaTCP6	ATCTTGGCTGTGTAGGATGGTGAA	GCATTCCTTCATTTGCCATCTAAC
PaTCP7	TCTGGGAGAAGAGACGACGATG	CGAATGCTAACGATTGAAATGACG
PaTCP8	GCCCACATTCCAATCCAGGT	CGGAACTGATGATGATCGACCAT
PaTCP9	GCAGTAGTCGCACCAAAACTCATC	GCATTGTTGGGGTGGGTAGTGT
PaTCP10	TGAAATGCCGAAGGCGAAGTC	GATTCGGCTGTCATTCAACTGC
PaTCP11	GCCGCTGGTCAGAGATGAAGAG	CATCTGCCTAATCCATCAAAACACA
PaTCP12a	AGGAAGCCAGTTCTCAATCGTTAG	ACATTGGGCATTGGGGTAAAGT
PaTCP12b	CGGTTTCAGTTCCAGCATTTAGAT	ACTGCCAGACTTTGCCATAATCCA
PaTCP13	GTCCTAACCTTTCCCACAACAATG	TCGGACTCCCTTCCATCACTAT
PaTCP14a	TACGAGTCATAACACGGGCGAT	ATGGTGGCTCGTTGTATCGTGC
PaTCP14b	GGAGCCCTCAACAAAAGCAA	GCGGTTCGGATTAGAGAAGACG
PaTCP15	ATTGGGAATGTTGACGGCGA	GCGTAATCTGCGAAGACTTTGAA
PaTCP17	AGGGCACAACTCAGTCATTTTCAT	CGTTTCTCAATAACACTTCCTCCAG
PaTCP18a	GCCCCATTTGCTCATCTTTATTCA	GGTCATTTCCACTGATTAAAGCGTC
PaTCP18b	GTGATGCTCAGCCAGAAGAATGT	TGCCCGTAGGAGTTTTGGCTTAGT
PaTCP18c	GCGGTAGTTCCATCCTTAGATAGTG	TGTTTGTGAACTTGCTGCTTTGAC
PaTCP19a	CGAACAGTGATGGAAGTGGAGATA	ACGATTTGGAAGAGGGACTTTGAT
PaTCP19b	ATGGTGGGAGGACCGTTAGTGA	GCGGCTTTGAAAATGTAACTTGAG
PaTCP20	CGATTAGTTTCCCTGGCTTTGA	CTCAGGAGAAGGCTGTTGATGCT
PaTCP21	CGGAGAAGAGACGACGATGGT	CGCTTATTGAGAGTTCTGTGCCTA
PaTCP22	CGTGGGTTAGGTGTTTCAGAGACT	CGCTCACTACCTTGTGTCTGGCTA
PaTCP23	CGGGTTACACTTTTCACACATTCAG	CACATCAATCCAAAGCCAGCAC
PaTCP24a	CGTCTACATCTAGTCCCCCCTACA	GCTTTTGAATCTCCTCCATCTCCA
PaTCP24b	CACCAAACCACGCCGAGACA	AGGATGATGGTCCCCTGTGATAC
PhEF1α	CCTGGTCAAATTGGAAACGG	CAGATCGCCTGTCAATCTTGG

B. Sequences of the primers for amplification of full-length CDS

Primer name	The forward primer sequence (5′-3′)	The reverse primer sequence (5′-3′)
PaTCP3a-CDS	CCCGTCTTCCTCGTCTCAAA	GGACTCTATGACAGCATTACTTCT
PaTCP4a-CDS	GATATCCACTGAATATGCAAAGAGT	CAGCAGCACAACTTAATTGACAA
PaTCP12b-CDS	CCACATTCTTTTCCTCTCATCAC	CAACTGCCAGACTTTGCCATA

C. The mature sequences of *PamiR319*.

miRNA	Mature sequence (5'-3')
PamiR319a-e	UUGGACUGAAGGGAGCUCCCU
PamiR319h, i	UUGGACUGAAGGGAGCUCCU
